# Vasculogenic mimicry, a negative indicator for progression free survival of lung adenocarcinoma irrespective of first line treatment and epithelial growth factor receptor mutation status

**DOI:** 10.1186/s12885-021-07863-z

**Published:** 2021-02-06

**Authors:** Xuejun He, Jijun You, Haibing Ding, Zhisheng Zhang, Lin Cui, Xiaomei Shen, Xiaoxia Bian, Yanqing Liu, Jue Chen

**Affiliations:** 1 Oncology Department, the Second People’s Hospital of Taizhou affiliated to Medical College of Yangzhou University, No. 27, Jiangyan District, Taizhou, 225500 China; 2 Orthopaedic Department, the Second People’s Hospital of Taizhou Affiliated to Medical College of Yangzhou University, Taizhou, China; 3 Institute of Medicine, Yangzhou University, No. 88, South Daxue Road, Yangzhou, 225001 China; 4 Respiratory Department, the Second People’s Hospital of Taizhou Affiliated to Medical College of Yangzhou University, Taizhou, China

**Keywords:** Lung adenocarcinoma, Epithelial growth factor receptor mutation, Prognosis, Treatment response, Vasculogenic mimicry

## Abstract

**Background:**

Vascular mimicry (VM) was associated with the prognosis of cancers. The aim of the study was to explore the association between VM and anticancer therapy response in patients with lung adenocarcinoma.

**Methods:**

This was a single-center retrospective study of patients with lung adenocarcinoma between March 1st, 2013, to April 1st, 2019, at the Second People’s Hospital of Taizhou City. All included patients were divided into the VM and no-VM groups according to whether VM was observed or not in the specimen. Vessels with positive PAS and negative CD34 staining were confirmed as VM. The main outcome was progression-free survival (PFS).

**Results:**

Sixty-six (50.4%) patients were male. Eighty-one patients received chemotherapy as the first-line treatment, and 50 patients received TKIs. Forty-five (34.4%) patients were confirmed with VM. There was no difference regarding the first-line treatment between the VM and no-VM groups (*P* = 0.285). The 86 patients without VM had a median PFS of 279 (range, 90–1095) days, and 45 patients with VM had a median PFS of 167 (range, 90–369) days (*P* < 0.001). T stage (hazard ratio (HR) = 1.37, 95% confidence interval (CI): 1.10–1.71), N stage (HR = 1.43, 95%CI: 1.09–1.86), M stage (HR = 2.85, 95%CI: 1.76–4.61), differentiation (HR = 1.85, 95%CI: 1.29–2.65), therapy (HR = 0.32, 95%CI: 0.21–0.49), VM (HR = 2.12, 95%CI: 1.33–3.37), and ECOG (HR = 1.41, 95%CI: 1.09–1.84) were independently associated with PFS.

**Conclusion:**

The benefits of first-line TKIs for NSCLC with EGFR mutation are possibly better than those of platinum-based regimens in patients without VM, but there is no difference in the benefit of chemotherapy or target therapy for VM-positive NSCLC harboring EGFR mutations.

## Background

Lung cancer is currently the predominant malignant disease with the highest cancer-related death rate worldwide [1–3]. In the past twenty years, with the discovery and better understanding of mutations in the epithelial growth factor receptor (EGFR) as a cancer-driving event and the use of tyrosine kinase inhibitors (TKIs), patients with lung cancer harboring EGFR-sensitizing mutation have benefited from prolonged progression-free survival (PFS) compared with patients who received conventional platinum-based chemotherapy only [4]. Nevertheless, most of the patients who receive first-generation TKIs will eventually progress after a median of 10–14 months [5]. Patients confirmed with progression after a standard first-line TKI therapy were proved to be responsive to second-line platinum-based chemotherapy. Data from the LUX-LUNG3/6 trials indicated that the overall survival (OS) benefit from TKIs followed by platinum-based chemotherapy vs. chemotherapy followed by TKIs was similar [6]. Therefore, it is considered that different mechanisms are underlying the response to chemotherapy and TKIs in patients with non-small cell lung cancer (NSCLC) harboring EGFR mutation, but which subpopulation will respond better to chemotherapy or TKIs is still unknown. In advanced NSCLC harboring EGFR-sensitive mutations, it has become a consensus that TKIs should be preferred for first-line treatment, but the effective response rates to first-generation TKIs such as gefitinib and erlotinib and the next-generation drugs such as afatinib and oxetinib are less than 80% [7, 8]. Therefore, how to predict whether NSCLC patients will respond effectively to first-line TKIs before starting treatment has become a key problem to be solved urgently.

Vascular mimicry (VM) was first described in human melanoma in 1999, followed by many other cancers such as esophageal carcinoma, hepatocellular carcinoma, gastric cancer, and lung cancer [9–11]. The presence of VM indicates shortened PFS and OS [12]. Some preclinical pharmacological research also demonstrated that VM is associated with resistance to anticancer treatment [13, 14]. Driving EGFR mutations lead to the downstream activation of the Ras/Raf/MAPK and PI3K-AKT-mTOR signaling pathways, which have been demonstrated as being key factors in cellular proliferation, apoptosis resistance, angiogenesis, and metastasis [15]. The activated Ras/Raf/MAPK and PI3K-AKT-mTOR signaling pathways play crucial roles in VM development [16, 17]. Hence, it can be hypothesized that TKIs will have higher response rates in treating EGFR-mutant lung adenocarcinoma harboring VM, but data is lacking.

Therefore, the aim of this single-center cohort study was to explore the association between VM and the response to anticancer therapy in patients with lung adenocarcinoma and to examine the difference between TKIs and platinum-based regimen as first-line therapy in EGFR-mutant lung adenocarcinoma with VM.

## Methods

### Patients

This was a single-center retrospective study of patients confirmed pathologically with lung adenocarcinoma between March 1st, 2013, to April 1st, 2019, at the Second People’s Hospital of Taizhou City, identified by searching the electronic medical record system (EMR). The inclusion criteria were: 1) age 18–80 years; 2) pathologically confirmed advanced lung adenocarcinoma, stage IIIB or IV; 3) EGFR sensitizing mutation; 4) without chemotherapy, radiotherapy, surgery, immunotherapy, or traditional Chinese medicine before diagnosis; 5) at least one measurable lesion according to Response Evaluation Criteria in Solid Tumors (RECIST, version 1.1); and 6) Eastern Cooperative Oncology Group performance status (ECOG PS) of 0–2. The exclusion criteria were: 1) survival ≤90 days after diagnosis; 2) serious existing physical illnesses (heart disease, hepatic disease, renal disease, or pulmonary disease); or 3) underwent radiotherapy directly after diagnosis (e.g., superior venous cava syndrome, spinal cord compression, or brain metastasis with obvious symptoms). All included patients were divided into the VM and no-VM groups according to whether VM was observed or not in the specimen.

The study was approved by the institutional review board of the second People’s hospital of Taizhou city (TZEYLL20121207001) and conducted according to the Declaration of Helsinki and Chinese laws. The need for individual consent was waived by the committee because of the retrospective nature of the study.

### Treatment

According to the earlier version of the practice guidelines from the Chinese Society of Clinical Oncology (CSCO), chemotherapy and/or TKIs were given as the first-line treatment for advanced lung adenocarcinoma [18]. In this analysis, platinum-based chemotherapy regimens included pemetrexed combined with carboplatin, docetaxel combined with cisplatin, and gemcitabine with cisplatin. The chemotherapy cycles lasted 3–4 weeks, and a maximum of six cycles was given unless progression was confirmed or intolerable side effects occurred. First-generation TKIs in this study were gefitinib and erlotinib. Drugs were administered until disease progression. For patients with early progression, second-line regimens were allowed.

### Data collection and follow-up

Data were collected from the EMR, including sex, age, smoking history, T stage, N stage, M stage, differentiation, EGFR mutation, ECOG PS, therapy, and survival. Paraffin-embedded tissues derived from biopsy before treatment were used to process mutation and VM assay. EGFR common mutations (exon 18, 19, 20, and 21) were identified using the amplification refractory mutation system (ARMS) and/or next-generation sequencing (NGS).

Two patterns of VM (matrix and tubular) were detected using PAS-CD34 dual staining. Tube-like structures with positive PAS and negative CD34 staining were confirmed as tubular VM. Besides, basement membrane surrounded by tumor cells were identified as matrix VM for positive PAS and negative CD34 staining [19, 20]. The presence of VM was determined in five randomized visual fields under a microscope. Tumor assessments were performed at baseline, after the 4th cycle, after the 6th cycle, and every 8 weeks until disease progression using computed tomography (CT) and/or magnetic resonance imaging (MRI). Response was classified as complete response (CR), partial response (PR), stable disease (SD), and progressive disease (PD) according to RECIST 1.1 [21].

The main outcome was PFS [21], which was defined as the duration from the first treatment administration to progression. The secondary outcomes were the objective response rate (ORR, CR + PR) and the disease control rate (DCR, CR + PR + SD) [22].

Follow-up data were collected from the EMR of the hospital. Follow-up was performed routinely every month during first-line treatment and every 3 months during the observation period. The last follow-up was on April 1st, 2019.

### Statistical analysis

All statistical analyses were performed using SPSS 19.0 for Windows (IBM, Armonk, NY, USA). Continuous variables were presented as means ± standard deviations and analyzed using the independent sample t-test. Categorical variables were presented as numbers and frequencies and compared the chi-square test. Pearson and Spearman correlation coefficients were used to determine the relationship between two variables. Survival analysis was performed using the Kaplan-Meier method and the log-rank test. Univariable and multivariable Cox regression models were used to analyze the association of variables with survival. Two-sided *P*-values < 0.05 were considered statistically significant.

## Results

### Characteristics of the patients

**Fig. 1:**
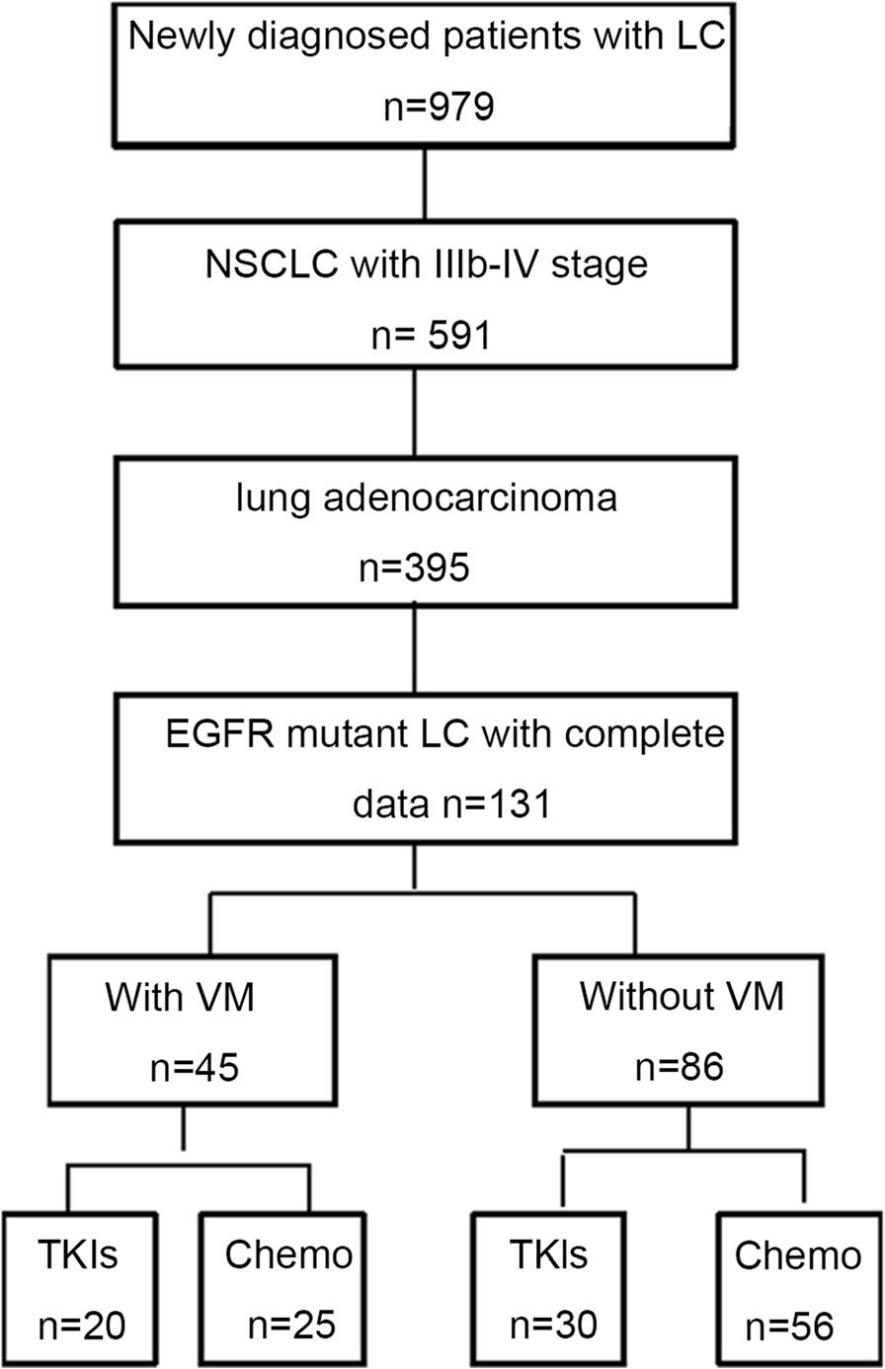
Patient flowchart. LC: lung cancer. NSCLC: non-small cell lung cancer. EGFR: epithelial growth factor receptor. VM, vasculogenic mimicry. TKIs: tyrosine kinase inhibitors. Chemo: chemotherapy

A total of 979 patients were newly diagnosed with lung cancer during the study period, of whom 591 patients were pathologically confirmed with NSCLC, and 395 patients were confirmed with lung adenocarcinoma of stage IIIB-IV. EGFR mutations were found in 153 patients. Finally, 131 patients were included in this study (Fig. 1). The median age was 63 (35–80) years. Sixty-six (50.4%) patients were male. Eighty-one patients received chemotherapy as the first-line treatment, and 50 patients received TKIs (Table 1).

**Table 1:** Demographic and clinical characteristics of the patients

Characteristics	Category	All (***n*** = 131)	With VM (***n*** = 45)	Without VM (***n*** = 86)	P
Sex	Male	66 (50.4)	23	43	0.904
	Female	65 (49.6)	22	43	
Age (years)	≤65	82 (62.6)	24	58	0.113
	> 65	49 (37.4)	21	28	
Smoking history	Without	97 (74.1)	32	65	0.579
	With	34 (26.0)	13	21	
T stage	T1	14 (10.7)	3	11	0.001
	T2	55 (42.0)	12	43	
	T3	36 (27.5)	17	19	
	T4	26 (19.9)	13	13	
N stage	N0	3 (2.3)	1	2	0.067
	N1	22 (16.8)	4	18	
	N2	52 (39.7)	14	38	
	N3	54 (41.2)	26	28	
M stage	M0	36 (27.5)	5	31	0.002
	M1	95 (72.5)	40	55	
Differentiation	Well	9 (6.9)	1	8	< 0.001
	Moderate	53 (40.5)	4	45	
	Poor	69 (52.7)	40	33	
EGFR-mutation	Exon 18	5 (3.8)	2	3	0.774
	Exon 19	55 (42.0)	18	37	
	Exon 20	8 (6.1)	2	6	
	Exon 21	63 (48.1)	23	40	
ECOG performance status	0–1	89 (67.9)	28	61	0.311
	2	42 (32.1)	17	25	
First-line therapy	chemo	81 (61.8)	25	56	0.285
	TKIs	50 (38.2)	20	30	

All are shown as n (%)

*VM* vasculogenic mimicry, *EGFR* epithelial growth factor receptor, *ECOG* Eastern Cooperative Oncology Group, *TKI* tyrosine kinase inhibitor

### Comparison of the VM and no-VM groups

**Fig. 2:**
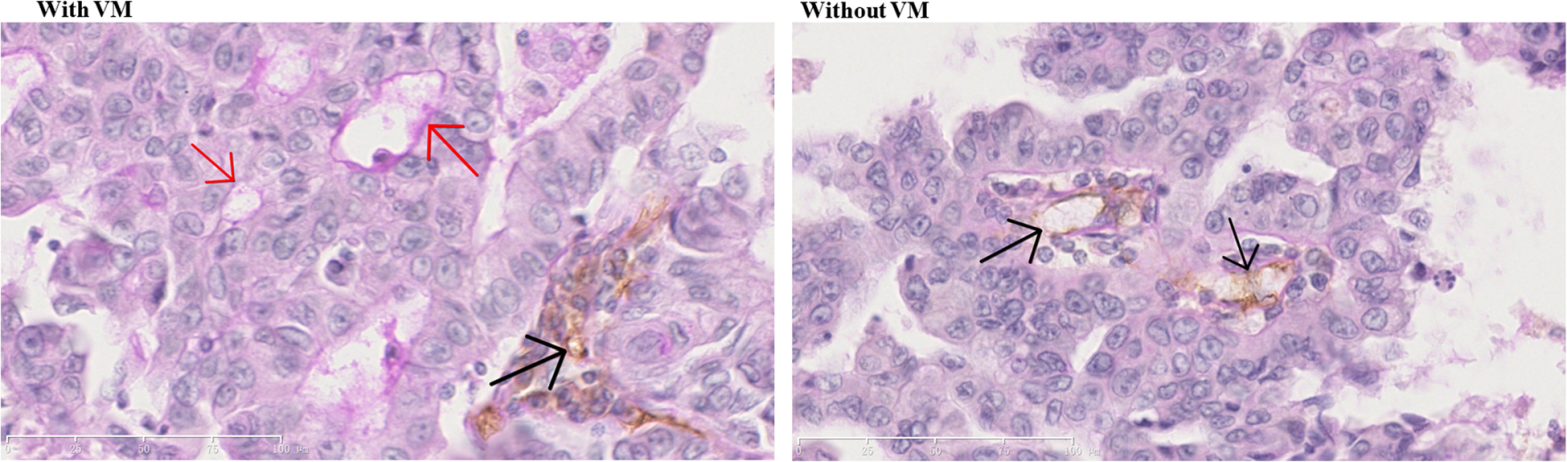
Typical matrix vasculogenic mimicry (VM) structure in lung adenocarcinoma. **a**: VM detected in one sample. **b**: a sample confirmed without VM. Red arrows indicate VM, and the black arrow indicates an endothelial vessel

A total of 45 (34.4%) patients were confirmed with the typical matrix VM structure (Fig. 2 and Table 1). Differentiation (*P* < 0.001), T stage (*P* = 0.001), and M stage (*P* = 0.002) were different between the two groups. There was no difference regarding the first-line treatment between the VM and no-VM groups (*P* = 0.285).

### Outcomes

**Fig. 3:**
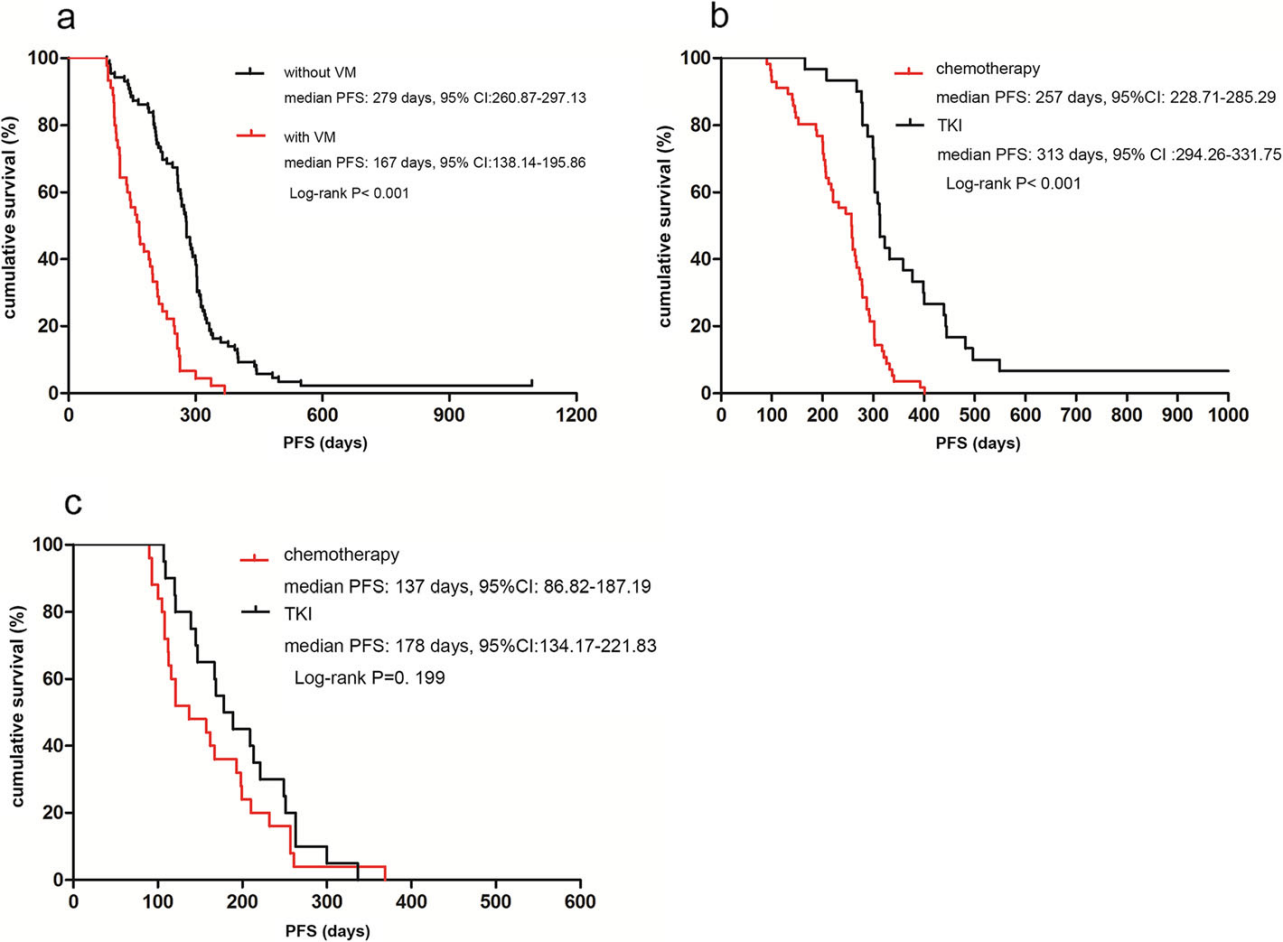
Survival analysis using the Kaplan-Meier method. **a** Median progression-free survival (PFS) of patients without VM was 279 days, compared was 167 days in patients with VM (log-rank test, *P* < 0.001). **b** In the no-VM group, the PFS of chemotherapy was 257 days (8.6 months) compared with 313 days (10.4 months) for TKIs (log-rank test, P < 0.001). **c** In the VM group, the PFS of chemotherapy was 137 days (4.6 months) compared with 178 days (5.9 months) for TKIs (log-rank test, *P* = 0.199)

The 45 patients with VM had a median PFS of 167 (range, 90–369) days, and 86 patients without VM had a median PFS of 279 (range, 90–1095) days (*P* < 0.001) (Fig. 3a).

**Fig. 4:**
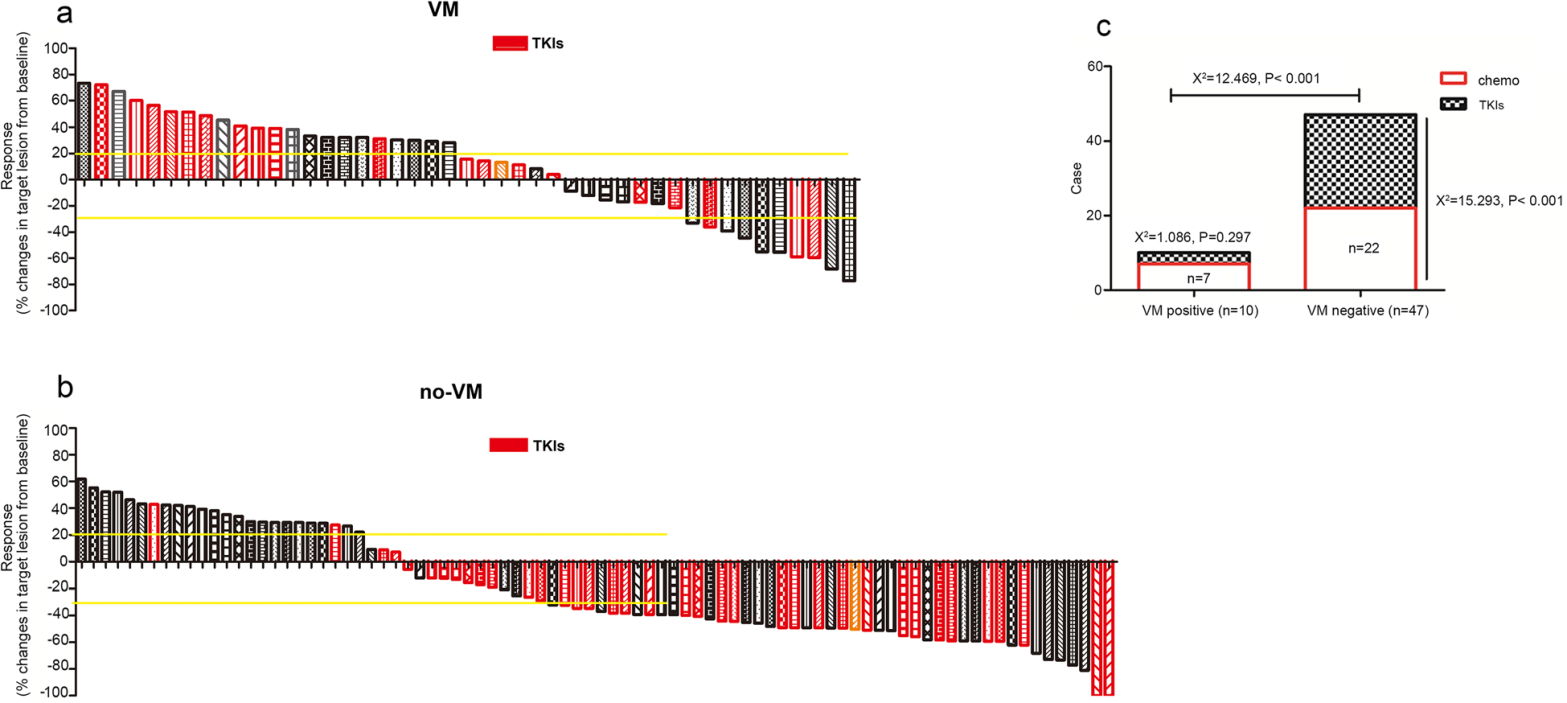
Waterfall graph of objective response rate (ORR), which was 22.2% in the vasculogenic mimicry (VM)-positive group (**a**) compared with 54.7% in the VM-negative group (**b**) (*P* < 0.001). In the VM-positive group, ORR of TKIs was 15% compared with 28% for chemotherapy (*P* = 0.297). In the VM-negative group, ORR of TKIs was 83.3% compared with 39.3% for chemotherapy (P < 0.001) (**c**). The red bar indicates chemotherapy

In the VM group, 10 patients achieved an objective response, including seven patients with chemotherapy and three patients with TKIs therapy. In the no-VM group, 47 patients achieved an objective response, including 22 patients with chemotherapy and 25 patients with TKIs therapy. The ORR was significantly different (*P* < 0.001) between the two groups. In the VM group, the ORR was similar between patients with chemotherapy and TKIs therapy. In the no-VM group, the ORR of patients with TKIs therapy was higher than for those with chemotherapy (P < 0.001) (Fig. 4).

### Univariable and multivariable analyses of factors associated with PFS

In the univariable analyses, age, smoking, T stage, N stage, M stage, differentiation, therapy, VM, and ECOG were associated with PFS (all *P* < 0.05). All the variables were included in the multivariable analysis. Then, T stage (hazard ratio (HR) = 1.37, 95% confidence interval (CI): 1.10–1.71), N stage (HR = 1.43, 95%CI: 1.09–1.86), M stage (HR = 2.85, 95%CI: 1.76–4.61), differentiation (HR = 1.85, 95%CI: 1.29–2.65), therapy (HR = 0.32, 95%CI: 0.21–0.49), VM (HR = 2.12, 95%CI: 1.33–3.37), and ECOG (HR = 1.41, 95%CI: 1.09–1.84 (were independently associated with PFS (all *P* < 0.05) (Table 2).

**Table 2:** Univariable and multivariable analyses of potential risk factors in advanced lung adenocarcinoma harboring EGFR mutation

Univariable analysis					Multivariable analysis			
Variable	HR	95.0% CI		P	HR	95.0% CI		P
Sex (male/female)	1.223	0.863	1.733	0.258	1.238	.851	1.801	0.263
Age (<65/≥65)	2.306	1.587	3.353	< 0.001	2.219	0.779	6.322	0.136
Smoking (never/smoking)	2.085	1.441	3.017	< 0.001	0.890	0.322	2.455	0.821
T stage (T1/T2–4)	1.537	1.264	1.869	< 0.001	1.374	1.103	1.712	0.005
N stage (N0/N1–3)	1.397	1.119	1.744	0.003	1.426	1.094	1.858	0.009
M stage (M0/M1)	1.685	1.137	2.497	0.009	2.847	1.761	4.605	< 0.001
Differentiation (non-poor/poor)	2.509	1.822	3.454	< 0.001	1.845	1.286	2.647	0.001
EGFR-mutation (Exon19,21/ Exon18,20)	1.065	0.899	1.261	0.467	1.152	.966	1.374	0.115
First-line therapy (chemo/TKIs)	0.431	0.292	0.635	< 0.001	0.321	0.210	0.492	< 0.001
VM (with/without)	3.406	2.299	5.047	< 0.001	2.118	1.331	3.372	0.002
ECOG (0–1/2)	1.394	1.096	1.773	0.007	1.412	1.085	1.837	0.010

*HR* hazard ratio, *CI* confidence interval, *VM* vasculogenic mimicry, *ECOG* Eastern Cooperative Oncology Group, *Chemo* chemotherapy, *TKIs* tyrosine kinase inhibitors

### Subgroup analysis of first-line therapy in the VM group

Among the 45 patients in the VM group, 25 patients received chemotherapy, and 20 received TKIs. The median PFS of chemotherapy was 137 (range, 90–369) days, and the median PFS of TKIs was 178 (range, 107–337) days (*P* = 0.199) (Fig. 3b). Among the 86 patients in the no-VM group, 56 patients received chemotherapy, and 30 received TKIs. The median PFS of chemotherapy was 257 (range, 90–401) days, and the median PFS of TKIs was 313 (range, 165–1095) days (*P* < 0.001) (Fig. 3c).

### VM reduced benefits from TKIs

**Fig. 5:**
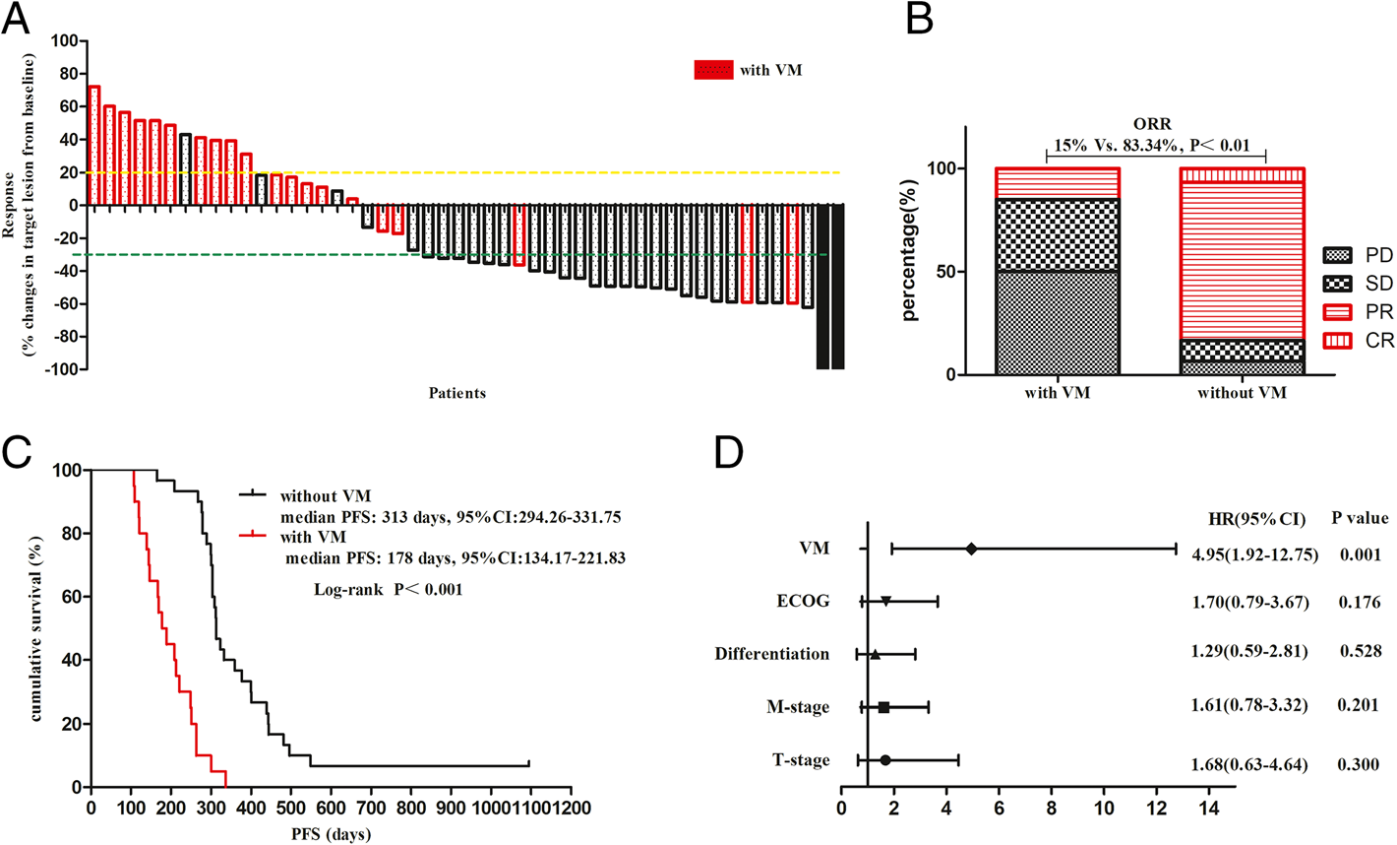
VM decreases benefits derived from first-line TKIs. **a** Waterfall graph of response rate in patients received TKIs, ORR in VM-positive patients was 15% compared with 83.34% for VM-negative (P < 0.001) (**b**). PFS was 313 (range, 165–1095) days in VM-negative patients compared with 178 (range, 107–337) days in VM-positive patients (P < 0.001) (**c**). VM was the independent factor on PFS (**d**)

As the outcomes being significant between VM and no-VM groups, we further analyzed ORR and PFS of patients receiving first TKIs with or without VM. In 50 patients receiving TKIs, 28 patients achieved an objective response, 25 of them were confirmed without VM and 3 confirmed with VM, a significant decline of ORR was observed in the presence of VM (Fig. 5 a, b). Patients receiving TKIs without VM had a median PFS of 313 (range, 165–1095) days, while patients with VM had a median PFS of 178 (range, 107–337) days (*P* < 0.001) (Fig. 5 c). As the COX analysis shows, VM was the independent factor on PFS irrespective of TNM stage and differentiation (Fig. 5 d).

## Discussion

The exact relationship between VM and anticancer therapy response in patients with lung adenocarcinoma is unknown. Therefore, this study aimed to explore the association between VM and the response to anticancer therapy in patients with lung adenocarcinoma and to examine the difference between TKIs and platinum-based regimen as first-line therapy in EGFR-mutant lung adenocarcinoma with VM. The results suggest that the benefits of first line TKIs for NSCLC with EGFR mutation are possibly better than those of platinum-based regimens in patients without VM, but TKIs seem to be no superior to chemotherapy in patients with VM.

The results showed that in advanced NSCLC patients harboring EGFR-sensitive mutations, first-line TKI therapy was superior to platinum-based chemotherapy in both ORR and PFS, which is consistent with the available data [23]. On the other hand, the PFS of patients with VM was significantly lower than that of no-VM, regardless of the first-line treatments. In the VM-positive subgroup, patients’ response to TKIs decreased, and PFS benefit from TKI treatment was not superior to that of chemotherapy. The data suggest that the presence of VM has a negative impact on the response to treatment and survival of lung adenocarcinoma with common EGFR mutations.

In the present study, the DCR was 76.0% (38/50) in the 50 NSCLC patients treated with first-line TKIs, which is consistent with the literature [24]. Among the 38 patients who achieved disease control, 10 (26.3%) were VM-positive; among the 12 patients with PD, 10 (83.3%) were VM-positive. These results suggest that VM-positive NSCLC patients have a lower response to TKIs. Generally, TKIs have shown promising applications in preclinical and clinical for NSCLC driven by common EGFR mutations [7, 8].

VM was observed in most malignant solid tumors, and the morbidity varies with the types of tumors [9–14]. The morbidity of VM reported in NSCLC was 28–42%, while it was 34.4% in this study. VM is a kind of functional blood vessel formed by high invasive tumor cells mimicking vascular endothelial cells. It has the function of supplying nutrients and oxygen and promoting tumor metastasis. Therefore, the presence of VM in tumors often suggests a higher degree of malignancy [9–14]. In the present study, VM was independently associated with PFS, along with tumor differentiation and TNM. The literature suggests that VM is associated with poor prognosis and survival [9–14].

At present, there are some reports about the mechanisms underlying VM and its value in evaluating clinical prognosis, but few reports about the effect of VM on the efficacy of therapy. Data from this study showed that the median PFS without VM was 10.4 months in patients with NSCLC receiving TKI first-line treatment, while it was 7.0 months among patients with VM. The median PFS in EGFR-mutant NSCLC with VM was shorter than that reported in the literature (9–12 months) [9–12], suggesting that VM may cause resistance to TKIs. The median PFS of the patients with VM was 3.9 months, significantly shorter than the 8.6 months of patients without VM. The results suggest that VM also may be one of the potential factors inducing resistance to chemotherapy. In addition, 27.4% of the patients with disease control in this study were VM positive, lower than 46.8% of patients having PD. Therefore, VM may be associated with primary resistance to first-line treatment in EGFR-mutant NSCLC.

Since Maniotis et al. [9] first described the morphology of VM in melanoma in 1999, numerous studies have been conducted to explore the underlying mechanisms. Hypoxia is one of the key conditions for VM development [25]. There are large numbers of hypoxia-inducible factors localized in the VM region [25, 26]. Hypoxia can directly regulate the expression of genes such as vascular endothelial growth factor-A, vascular endothelial growth factor receptor-1, EphA2, Twist, Nodal, osteopontin (OPN), and COX-2, and directly promote the formation of VM [25, 26]. In addition, hypoxia is also an important factor leading to resistance to cancer treatment [27]. Recent studies confirmed that hypoxia could mediate the up-regulation of tissue protein deacetylase LSD1 and PLU-1, and promote the resistance to gefitinib in NSCLC with EGFR activating mutation [28]. Therefore, hypoxia, VM, and resistance to cancer therapy may be a sequential cascade of events. Of course, besides hypoxia, the phenotypic transformation of tumor cells is also an important factor in the formation of VM [29]. Previous studies have shown that activation of multiple signaling pathways related to EMT is involved in VM development [29–31]. A previous study by our group also confirmed that the up-regulation of Notch1 promotes EMT and further induces VM formation in hepatocellular carcinoma [32]. In addition, EMT is a factor of treatment resistance in NSCLC [33, 34]. Therefore, we can reasonably believe that VM reflects the highly heterogeneous changes of tumor cells and can be regarded as an important marker of resistance to primary treatment in NSCLC.

In VM-positive patients with NSCLC and EGFR mutation, which treatment is more effective is still unknown. Nevertheless, in this study, we found that chemotherapy seems to achieve better disease control than TKIs for VM-positive NSCLC. Nevertheless, PFS in patients who received chemotherapy was similar to that of TKIs in VM-positive patients. This finding indicates that there is no difference in the benefit of chemotherapy or target therapy for VM-positive NSCLC harboring EGFR mutations. Whether anti-angiogenesis therapy could improve the prognosis of patients with VM remains to be determined.

Now in the world, especially in developing countries, the public health burden caused by cancer is relatively heavy [35]. However, because of the advantages of targeted therapy in perfect disease control and disease-free survival in lung adenocarcinoma, many guidelines still recommend TKIs as the first-line treatment in EGFR mutated NSCLC [36]. If the benefits of ORR and PFS derived from TKIs lost due to the heterogeneity of tumor, the dominant position of chemotherapy is still worth considering [37]. This study found that the presence of VM significantly reduced ORR and PFS of TKIs, even worse than chemotherapy in disease control. Considering the cost-benefit of tumor treatment, we believe that chemotherapy should be given priority in the treatment of lung adenocarcinoma with VM. In theory, chemotherapy or targeted therapy combined with anti-VM should be a good strategy for this kind of disease. However, at present, anti-VM only stays at the stage of preclinical pharmacology and early clinical research, and there is no reliable evidence to support anti-VM [38]. It is expected that more research will focus on this field, so that anti-VM combined with current strategies can bring promising benefits to such patients.

The present study has limitations. It was a single-center study with a relatively small sample size. In addition, its retrospective nature prevented the analysis of variables that are not routinely collected in the EMR. In addition, only common EGFR mutations and rare EGFR mutations were not analyzed, and their relationship with VM remains unknown.

## Conclusions

In conclusion, the benefits of first generation TKIs for NSCLC with EGFR mutation are possibly better than those of platinum-based regimens, but first generation TKIs seem to be no superior to chemotherapy in patients with VM. Whether chemotherapy combined with targeted therapy or anti-VM therapy has greater clinical benefits needs to confirmed in future studies.

## Data Availability

The datasets used and/or analysed during the current study are available from the corresponding author on reasonable request.

## Abbreviations

VM: Vascular mimicry
EGFR: Epithelial growth factor receptor
TKIs: Tyrosine kinase inhibitors
PFS: Progression-free survival
OS: Overall survival
NSCLC: Non-small cell lung cancer
EMR: Electronic medical record system
CSCO: Chinese Society of Clinical Oncology
NGS: Next-generation sequencing
CT: Computed tomography
MRI: magnetic resonance imaging
CR: complete response
PR: Partial response
SD: Stable disease
ORR, CR + PR: Objective response rate
DCR, CR + PR + SD: Disease control rate
PD: Progressive disease
